# Personal traits and coping strategies in compliance with COVID-19 preventive measures

**DOI:** 10.1192/j.eurpsy.2021.807

**Published:** 2021-08-13

**Authors:** M. Kiseleva

**Affiliations:** Institute Of Psychosocial Work. Department Of Pedagogy And Medical Psychology, Federal State Autonomous Educational Institution of Higher Education I.M. Sechenov First Moscow State Medical University of the Ministry of Health of the Russian Federation (Sechenov University), Moscow, Russian Federation

**Keywords:** big five, coping strategies, pandemic, coronavirus (Covid-19).

## Abstract

**Introduction:**

There is no much data on the psychological predictors of compliance with Covid-19 preventive measures (self-isolation, social distancing, etc.), that are one of the most effective ways to combat the spread of the disease

**Objectives:**

This study is aimed to examine the role of personal traits in compliance with the Covid-19 preventive measures, and to identify the psychological features of those who are unmotivated to comply with quarantine.

**Methods:**

The study involved 256 participants aged from 16 to 73 years from Russia, and was conducted in March-April 2020.The coping strategies questionnaire (COPE), Big five personality questionnaire were used.

**Results:**

Compliance with the coronavirus preventive measures is positively associated with such personality traits as conscientiousness and friendliness, and productive coping strategies (active coping, planning, positive reformulation, acceptance). Another significant predictors of compliance with restrictive measures are explaining the reasons for the spread of coronavirus by lack of responsibility of people and violation of quarantine measures (positive predictor), as well as downplaying the risk of the disease (negative predictor).

**Conclusions:**

Cluster analysis identified two most common motivational profiles: unmotivated and motivated.Unmotivated people are less willing to comply with Covid-19 preventive measures. Unmotivated people trust various sources of information less often, use unproductive coping strategies (denial), and are more likely to believe that the coronavirus is used to hide the presence of other problems in the society and to increase the control over citizens by the state.

